# Supplementary figures and images for: Discovery and Functional Annotation of Quantitative Trait Loci Affecting Resistance to Sea Lice in Atlantic Salmon
Source: Front Genet. 2019 Feb 8;10:56. doi: 10.3389/fgene.2019.00056 (PMC6375901; doi:10.3389/fgene.2019.00056)

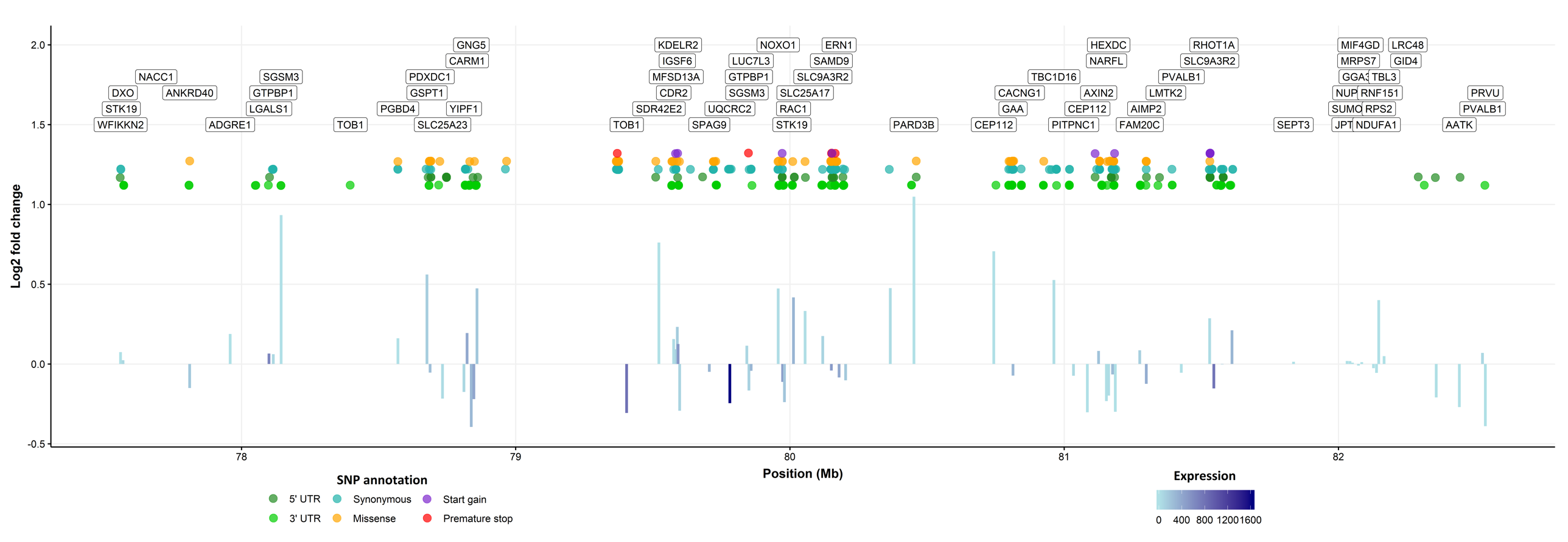

Supplement: FIGURE S1 — QTL region in chromosome 3. Bars represent the log 2 fold change between healthy and sea lice attachment skin for every gene in the QTL region according to the RNA-seq. Bar colour represents the expression level of the gene (lighter = less expressed), and the annotation of the gene is presented in a label on the top of the graph. Genic SNPs detected by WGS are shown in between, those with putatively more severe effects are located towards the top of the figure. [file Image_1.PNG]

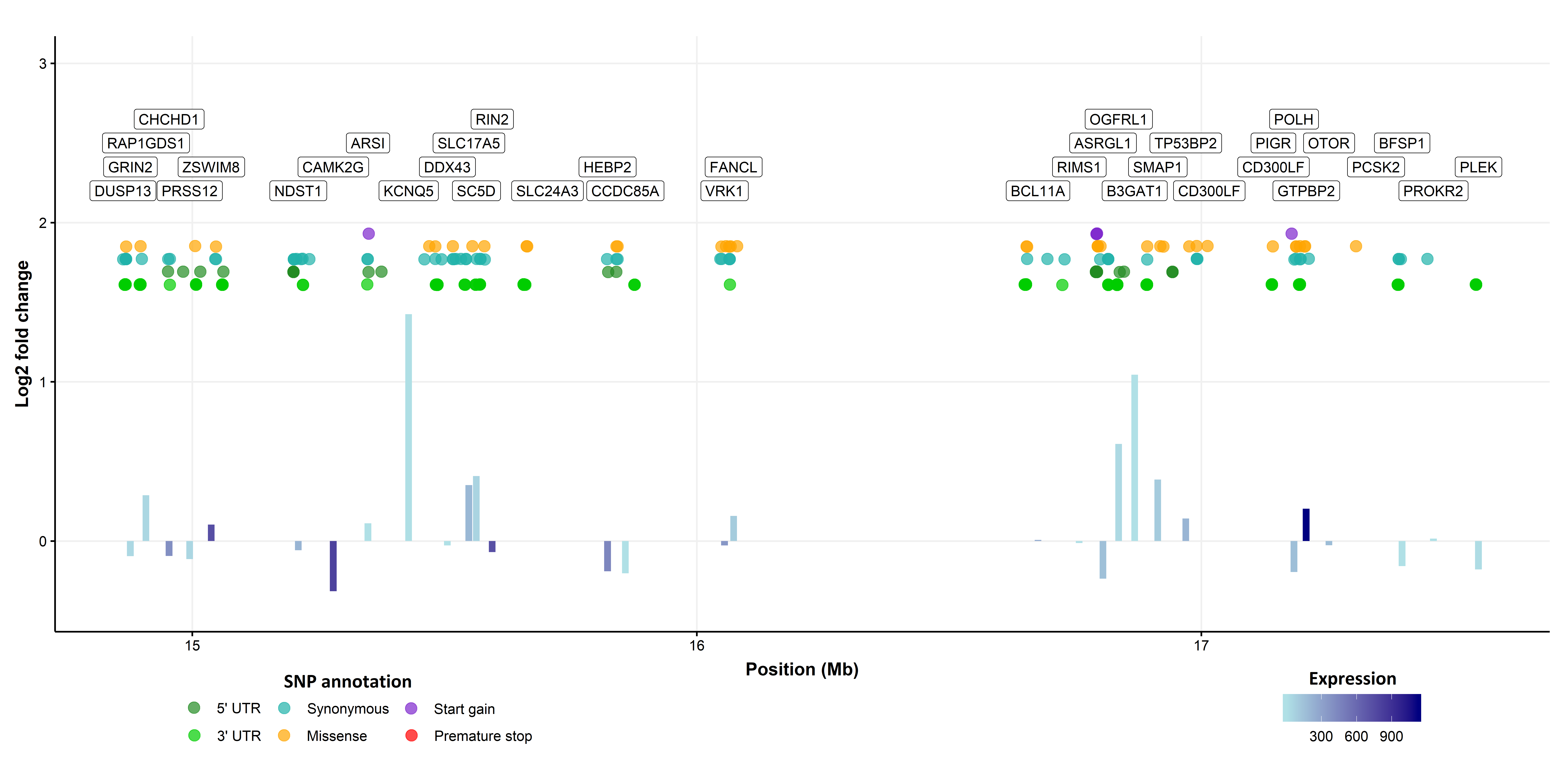

Supplement: FIGURE S1 — QTL region in chromosome 18. Bars represent the log2 fold change between healthy and sea lice attachment skin for every gene in the QTL region according to the RNA-seq. Bar colour represents the expression level of the gene (lighter = less expressed), and the annotation of the gene is presented in a label on the top of the graph. Genic SNPs detected by WGS are shown in between, those with putatively more severe effects are located towards the top of the figure. [file Image_2.PNG]
